# Effects of aeration modes and rates on nitrogen conversion and bacterial community in composting of dehydrated sludge and corn straw

**DOI:** 10.3389/fmicb.2024.1372568

**Published:** 2024-03-12

**Authors:** Yuyun Wang, PengXiang Xu, Yue Wang, Jing Su, Zhi Xu, Zhengbo Jiang, Yuquan Wei, Sheng Hang, Xiaoyan Ding, Hao Zhang, Longli Zhang, Yongdi Liu, Ji Li

**Affiliations:** ^1^College of Resources and Environmental Science, Yunnan Agricultural University, Kunming, China; ^2^Beijing Key Laboratory of Biodiversity and Organic Farming, College of Resources and Environmental Science, China Agricultural University, Beijing, China; ^3^Organic Recycling Institute (Suzhou) of China Agricultural University, Suzhou, China; ^4^Academy of Agricultural Planning and Engineering, Ministry of Agriculture and Rural Affairs, Beijing, China; ^5^Nanjing Institute of Environmental Sciences, Ministry of Ecology and Environment, Nanjing, China; ^6^Technical Centre for Soil, Agriculture and Rural Ecology and Environment, Ministry of Ecology and Environment, Beijing, China; ^7^Beijing VOTO Biotech Co., Ltd., Beijing, China

**Keywords:** composting, aeration modes, aeration rates, nitrogen fractions conversion, NH3 emission

## Abstract

Aeration is an important factor to regulate composting efficiency and nitrogen loss. This study is aimed to compare the effects of different aeration modes (continuous and intermittent) and aeration rate on nitrogen conversion and bacterial community in composting from dehydrated sludge and corn straw. Results showed that the intermittent aeration mode at same aeration volume was superior to the continuous aeration mode in terms of NH_3_ emission reduction, nitrogen conversion and germination index (GI) improvement. Intermittent aeration mode with 1200 L/h (aeration 5 min, stop 15 min) [K5T15 (V1200)] and 300 L/h of continuous aeration helped to the conservation of nitrogen fractions and accelerate the composting process. However, it was most advantageous to use 150 L/h of continuous aeration to reduce NH_3_ emission and ensure the effective composting process. The aeration mode K5T15 (V1200) showed the fastest temperature rise, the longer duration of thermophilic stage and the highest GI (95%) in composting. The cumulative NH_3_ emission of intermittent aeration mode was higher than continuous aeration mode. The cumulative NH_3_ emission of V300 was 23.1% lower than that of K5T15 (V1200). The dominant phyla in dehydrated sludge and corn straw composting were Firmicutes, Proteobacteria, Actinobacteria, and Bacteroidetes. The dominant phylum in the thermophilic stage was Firmicutes (49.39%~63.13%), and the dominant genus was *Thermobifida* (18.62%~30.16%). The relative abundance of Firmicutes was greater in the intermittent aeration mode (63.13%) than that in the continuous aeration mode (57.62%), and *Pseudomonas* was dominant in composting with lower aeration rate and the lowest NH_3_ emission. This study suggested that adjustment to the aeration mode and rate could affect core bacteria to reduce the nitrogen loss and accelerate composting process.

## 1 Introduction

There are over 600 million tonnes of corn straw produced each year in China, ranking first in the world (Liu et al., 2021). Corn straw is rich in organic matter, cellulose, crude protein, crude fat and various nutrients such as nitrogen, phosphorus, potassium, calcium, magnesium, etc., which can be seen as resources for recycling. On the other hand, the production amount of urban and industrial sewage in China had reached 7.34 × 10^8^ t/m^2^, and there is over 7.29 × 10^5^ t/m^2^ after dehydration. The large quantities of dehydrated sludge and straw have gradually become a social problem, which may lead to environmental pollution and hinder economic development (Zhou et al., 2023). Composting is an effective way for dehydrated sludge and corn straw treatment through microbial aerobic metabolic activity, which is conducive to produce nontoxic and nutrient-rich organic fertilizers to improve soil fertility, and enhance crop yield (Zhao et al., 2024).

Nitrogen, as an important element in organic wastes, provides an important nutrient for microbial growth and ensures the smooth progress of composting (Caceres et al., 2015). It is widely reported that raw material properties, and process control parameters are the main factors affecting microbial activity in composting, thus affecting nitrogen conversion and ammonia emission (Meng et al., 2016). Ammonia (NH_3_) is one of the main odors produced in composting and NH_3_ emission was the main factor of nitrogen loss in composing, leading to the reduction of the agronomic quality of products (Shan et al., 2021). NH_3_ is volatilized from NH_4_^+^-N by the ammonification of degradable organic nitrogen at optimum pH and higher temperature (Meng et al., 2016; Han et al., 2018). NH_3_ emission in composting is significantly affected by many factors such as temperature, moisture content, aeration modes, pH, etc. (Manu et al., 2021). Among these factors, many studies indicated that aeration is the largest contributor to regulate NH_3_ emission but excessive aeration may result in more nitrogen loss (Jiang et al., 2015).

Forced aeration is widely used in large-scale composting plants and it is crucial to control the aeration mode and rate for composting efficiency, product quality, gas emissions, and operation costs (Hoang et al., 2022). However, the aeration rate usually varied depending on the raw materials (Gao et al., 2010). Keener et al. (2001) concluded that aeration rates of 0.3 ~ 0.9 L/(min **·** kg organic matter) were required for agricultural wastes. For municipal waste, it is suggested that the aeration rate of 0.06 ~ 0.4 L/(min **·** kg organic matter) is more reasonable (Xiong et al., 2017). A large number of studies have also shown that the required aeration rates were different for varied composting process conditions. Rasapoor et al. (2009) showed that the aeration rate at 0.6 L/(min **·** kg organic matter) at the initial stage of composting was the most reasonable for the municipal wastes and was better to decrease to 0.4 L/(min **·** kg organic matter) at the maturation stage of composting. Therefore, it can be concluded that the aeration rate parameters are appropriate in the range of 0.2 ~ 0.6 L/(min **·** kg organic matter) (Chowdhury et al., 2014; Talib et al., 2014; Zhang et al., 2016; Wu et al., 2019). However, there is also an obvious effect of aeration mode on the nitrogen conversion and loss in composting (Peng et al., 2023; Lai et al., 2024). It is reported that intermittent aeration can reduce cumulative NH_3_ emission and total nitrogen loss compared with continuous aeration, but increase N_2_O accumulation by the alternation of nitrification and denitrification (Wang et al., 2021). However, most studies focused on the effect of aeration mode and rate on compost product and gas emission. Considering that microbial community is the driving factor of composting process, it is urgent to pay attention to the interaction relationship between aeration, nitrogen fractions conversion and microbial community.

In this study, different aeration modes (continuous and intermittent) and rates were compared on the dehydrated sludge and corn straw composting. Composting basic physico-chemical performances, NH_3_ emission, and nitrogen conversion were assessed and the succession of bacterial community was investigated based on high-throughput sequencing. This study helps to understand the potential microbiological mechanism of the effect of aeration modes and rates on nitrogen conservation in composting.

## 2 Materials and methods

### 2.1 Raw materials

The raw materials used in the experiment were dehydrated sludge and corn straw. The dehydrated sludge was taken from Xiaojiahe wastewater treatment plant in Haidian District, Beijing. The corn straw came from Shangzhuang experimental station of China Agricultural University. The characteristics of raw materials were shown in Table 1. The dehydrated sludge and corn straw were thoroughly mixed in a ratio of 1:2 (volume), resulting in a carbon nitrogen ratio of 22. Each batch of mixture was divided in proportion in each reactor, ensuring the consistency of the raw material composition in composting.

**Table 1:** Characteristics of raw materials used for the composing experiment.

Raw materials	Moisture content (%)	Organic carbon (% DM)	Total nitrogen (% DM)	carbon/nitrogen (%)	pH value
Dehydrated sludge	80.7 ± 0.9	24.6 ± 0.01	3.7 ± 0.0	6.7	7.4 ± 0.1
Corn straw	11.4 ± 0.9	39.8 ± 0.1	0.5 ± 0.0	73.9	6.3 ± 0.0

### 2.2 Experimental system and protocol

The composting device used in this study is a set of small closed composting reactor system, which is composed of air compressor, thermal insulation fermentation tank, exhaust fan, thermometer and air volume controller. The effective volume of the reactor was 100 L and the size was 55 cm × 80 cm × 60 cm. The main body of the reactor is a closed silo-type fermentation tank, ventilated by air compression pump to ensure that the oxygen inside the reactor is not less than 10%. The air is forced into the reactor from the bottom of the reactor, and the aeration rate is ranged from 0 to 1,500 L/h, which can be precisely controlled by adjusting the aeration volume with glass rotor flowmeter. A centrifugal fan is arranged at the top of the reactor to discharge the water and gas in time. There is an electronic thermometer inside the reactor, which can monitor the temperature change in real time.

The aeration modes included continuous aeration and intermittent aeration. Six aeration rates were set up for continuous aeration experiment, that is, 150, 300, 600, 900, 1,200, and 1,500 L/h, which were named as V150, V300, V600, V900, V1200, and V1500, respectively. Four aeration rates were set up for the intermittent aeration experiment with same amount of gas entering each reactor in each 20 min, which were 300 L/h (continuous aeration, no stop), 400 L/h (aeration 15 min, stop 5 min), 600 L/h (aeration 10 min, stop 10 min), and 1,200 L/h (aeration 5 min, stop 15 min), which were defined as K20T0 (V300), K15T5 (V400), K10T10 (V600), and K5T15 (V1200), respectively. Three replicate trials were set up for all treatments.

Composting samples (500 g) were collected on day 0, 2, 4, 6, 8, 10, and 12, and each sample was collected using the multi-point sampling method. Part of samples were stored in the refrigerator at −20°C, and the remaining samples were air-dried and ground through 0.2 mm sieve and stored in a sealed container.

### 2.3 Analysis methods

The temperature was measured by an electronic thermometer. The O_2_ and NH_3_ content were tested using a combination gas detector. The moisture content (MC), pH, and germination index (GI) were determined with reference to the Chinese National Standard (NY 525-2021). The organic carbon and total nitrogen were tested by an element analyzer (Sheng et al., 2023). The nitrate nitrogen content, ammonium nitrogen content, and amide nitrogen content were measured with the reference to the Chinese National Standard (NY/T 1116-2014). The organic nitrogen content was determined by differential subtraction method.

The bacterial community analysis was performed via high-throughput 16S rRNA gene pyrosequencing as described by Wei et al. (2018). The total DNA of bacterial community was extracted by soil DNA kit (Omega Biotek, Inc.). The PCR amplification of 16S rRNA gene fragments was performed based on the universal primers 515F (5′-GTGCCAGCMGCCGCGGTAA-3′) and 909R (5′-CCCCGYCAATTCMTTTRAGT-3′). High-throughput sequencing for the purified 16S rRNA gene fragments were performed using the Illumina sequencing platform of Hiseq2500 by Novogene (Beijing, China). The sequences were submitted to the NCBI (PRJNA730304).

Multivariate analysis was conducted using SPSS 22 for one-way ANNOVA (Gao et al., 2019). The bacterial community data were analyzed using the tools in a galaxy instance^1^ (Zhan et al., 2021). Microsoft Excel 2016 was used for data analysis and Origin 2021 was used for graph production.

## 3 Results and discussion

### 3.1 Changes in physiochemical characteristics

**Figure 1:**
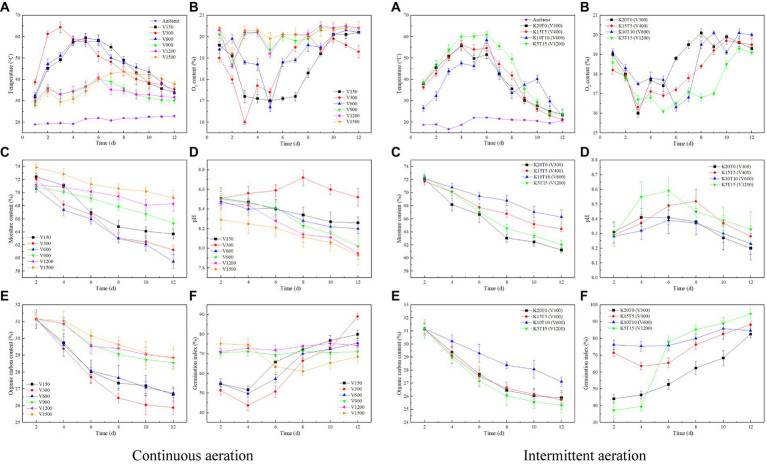
Variations of temperature **(A)**, O_2_ content **(B)**, moisture content **(C)**, pH **(D)**, organic carbon content **(E)**, germination index **(F)** at different aeration rates of continuous (left) and intermittent (right) aeration mode during composting.

The different aeration rates had a large effect on the variation of composting temperature under both continuous aeration and intermittent aeration modes (Figure 1A). When the aeration rate was less than 600 L/h by continuous aeration, the temperature increased firstly and then decreased. The temperature of V300 increased faster than other groups and achieved the highest temperature (64.4°C). When the aeration rate was greater than 900 L/h by continuous aeration, the rise of temperature was difficult, and the maximum temperature was only 40°C, leading to an incomplete thermophilic stage. Thus, it can be found that the suitable aeration rate was in the range of 150 ~ 600 L/h in a small closed reactor under continuous aeration mode and 300 L/h was the best aeration rate. As for intermittent aeration mode, the temperature of K20T0 (V300), K15T5 (V400), and K5T15 (V1200) peaked on day 4, and the peak value in order was K5T15 (V1200) > K15T5 (V400) > K20T0 (V300) (Figure 1A). The temperature of K5T15 (V1200) raised above 60°C and increased faster than other treatments and had the longer duration of thermophilic stage. In terms of temperature, the intermittent aeration mode of K5T15 (V1200) was better than others. Given the cumulative temperature and the raise rate of temperature, continuous aeration at 300 L/h and intermittent aeration of K5T15 (V1200) were more suitable for composting reactor. Since V300 of continuous aeration and K20T0 (V300) of intermittent aeration were the same aeration mode and rate, intermittent aeration was more conducive than continuous to the increase of composting temperature.

The O_2_ content is a direct indicator reflecting the intensity of aerobic fermentation reaction (Wu et al., 2024). The O_2_ content of each treatment in composting showed a trend of firstly decreasing and then increasing in both continuous aeration and intermittent aeration modes (Figure 1B). The O_2_ content decreased more on days 2–8 as the change in temperature, when the aeration rate was less than 600 L/h of continuous aeration. When the aeration rate was greater than 900 L/h of continuous aeration, the O_2_ content decreased very little. As for intermittent aeration, O_2_ consumption increased after day 4 in the thermophilic stage of composting and the lowest value of O_2_ content was 16.3% in K10T10 (V600). The O_2_ content of all treatments was greater than 16%, suggesting a sufficient oxygen supply for composting microbial metabolic activity (Mu et al., 2024).

During the composting process, the removal of moisture is not only related to the aeration rate, but also to the temperature. As the composting process progressed, the moisture content in each treatment showed a gradual decrease (Figure 1C). By the end of composting, the moisture content of V150, V300, V600, V900, V1200, and V1500 with continuous aeration decreased by 12.07, 15.13, 15.78, 7.83, 4.07, and 6.31%, respectively (*p* < 0.01). The moisture content removal rate was highest for V600, followed by V300 of continuous aeration, suggesting that relatively higher aeration rate helped to bio-drying. When the aeration rate was greater than 900 L/h of continuous aeration, the moisture content removal rate was lower due to more heat loss (Xin et al., 2023). As for intermittent aeration, the moisture content of K20T0 (V300), K15T5 (V400), K10T10 (V600), and K5T15 (V1200) decreased by 15.13, 10.16, 8.07, and 14.29%, respectively, after composting (*p* < 0.01). The above results suggested that continuous aeration with V600 and V300 produced a higher water removal rate for reactors of dehydrated sludge and corn straw composting.

The variations of pH values varied among the treatments and showed a trend of increasing and then decreasing in composting, especially in groups with intermittent aeration (Figure 1D). The increase of pH may be due to the accumulation of ammonia (Onwosi et al., 2017). The pH value of all groups ranged from 7.9 to 8.7, and composting with intermittent aeration (*p* > 0.05) had a more stable pH value in products compared to that with continuous aeration (*p* < 0.01).

The organic carbon content of each treatment showed a gradually decreasing trend (Figure 1E). When the aeration rate was less than 600 L/h with continuous aeration, the organic carbon content decreased more. By the end of composting, the organic carbon of V150, V300, and V600 with continuous aeration decreased by 14.35, 16.94, and 14.07%, respectively (*p* < 0.01). As for intermittent aeration, the organic carbon content K20T0 (V300), K15T5 (V400), K10T10 (V600), and K5T15 (V1200) decreased by 16.94, 17.40, 12.99, and 18.77%, respectively (*p* < 0.05). Overall, the organic carbon content degradation rate was the highest for V300 with continuous aeration and K5T15 (V1200) with intermittent aeration.

The GI value is an important index reflecting the harmless process of composting (Gao et al., 2019; Wei et al., 2020), which was gradually increasing in all groups in this study (Figure 1F). By the end of composting, the GI value of V150, V300, and V600 with continuous aeration were 79.94, 88.97, and 75.48%, respectively, meeting the requirement of organic fertilizer standard in China (>70%) (*p* < 0.01). As for intermittent aeration, GI value of K20T0 (V300) increased from 44.04% on day 2 to 82.52% at the end of composting, and K15T5 (V400), K10T10 (V600) and K5T15 (V1200) increased to 88.18, 84.68 and 94.65%, respectively (*p* < 0.01). Overall, the GI value of all treatments with intermittent aeration and V300 of continuous aeration reached more than 80% at the end of composting, with the highest GI value in K5T15 (V1200). As with the result of temperature, intermittent aeration was more conducive to the increase of GI value.

### 3.2 Changes in nitrogen conversion

**Figure 2:**
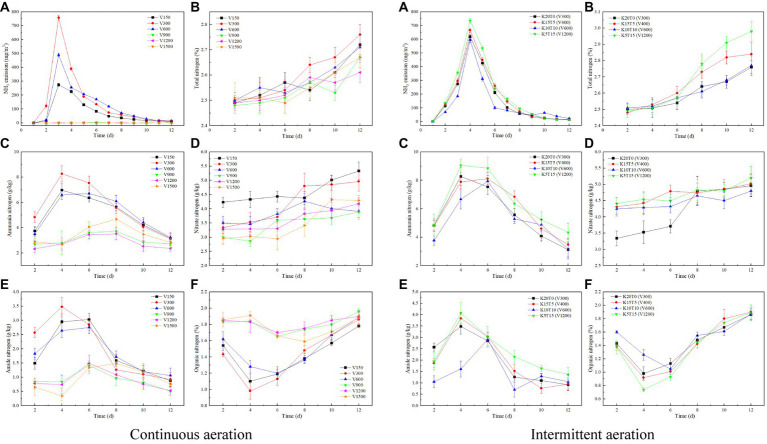
Variations of NH_3_ emission **(A)**, total nitrogen **(B)**, ammonia nitrogen **(C)**, nitrate nitrogen **(D)**, amide nitrogen **(E)**, and organic nitrogen **(F)** at different aeration rates of continuous (left) and intermittent (right) aeration mode during composting.

The NH_3_ emission could be ascribed to the intensive mineralization of organic nitrogen to NH_4_^+^, which was further transformed into NH_3_ under high temperature and alkaline conditions (Xiong et al., 2023). As the composting processed, the NH_3_ emission showed a trend of first rising and then falling (Figure 2A). The NH_3_ emission of V150, V300, and V600 of continuous aeration all reached the highest value on day 3 but the NH_3_ emission released from the pile was small for the treatments with the aeration rates greater than 900 L/h. As for intermittent aeration, K20T0 (V300), K15T5 (V400), K10T10 (V600), and K5T15 (V1200) reached the highest NH_3_ emission on day 4, with 619.3, 665.6, 595.0, and 736.9 mg/m^3^. Combined with the variation of pH value, the alkaline systems were more conductive to NH_3_ production. The cumulative NH_3_ emission of V150, V300, V600, V900, V1200, and V1500 of continuous aeration were 878.4, 1828.6, 1439.3, 13.3, 6.1, and 2.1 mg/m^3^, respectively. The cumulative NH_3_ emission of K20T0 (V300), K15T5 (V400), K10T10 (V600), and K5T15 (V1200) of intermittent aeration were 1910.6, 2101.1, 1576.7, and 2376.5 mg/m^3^, respectively. The cumulative NH_3_ emission of intermittent aeration mode was higher than continuous aeration mode. The cumulative NH_3_ emission of V300 was 23.1% lower than that of K5T15 (V1200).

The total nitrogen content of all treatments was about 2.50% at the beginning of composting and showed a gradual increasing trend (Figure 2B). By the end of composting, the total nitrogen content of V150, V300, V600, V900, V1200, and V1500 with continuous aeration increased by 9.24, 10.40, 8.40, 7.66, 4.82, and 6.37%, respectively, and V300 had the highest total nitrogen content at the end of composting (*p* < 0.05). As for intermittent aeration, the total nitrogen content of K20T0 (V300), K15T5 (V400), K10T10 (V600), and K5T15 (V1200) were 2.76, 2.84, 2.77, and 2.98% at the end of composting, and K5T15 (V1200) had the highest total nitrogen content in all groups (*p* < 0.01). The above results suggested that intermittent aeration mode by K5T15 (V1200) had more nitrogen conservation in composting.

Macromolecular organic matter was decomposed into small molecular substances such as amino acids, and the converted into ammonia nitrogen by ammoniated bacteria (Lehtovirta-Morley et al., 2013). The ammonia nitrogen content of all treatments showed a trend of increasing and then decreasing (Figure 2C). The ammonia nitrogen content of treatments with aeration rates less than 600 L/h with continuous aeration was higher than that of treatments with aeration rates greater than 900 L/h. The ammonia nitrogen content of V150 and V300 with continuous aeration reached the highest value on day 4, which were 6.97 g/kg and 8.27 g/kg, respectively, and the ammonia nitrogen content of V300 was higher than that of all other treatments. Combined with the variations of temperature, these results indicated that the thermophilic stage had higher ammonia nitrogen production than other stages (*p* < 0.05). As for intermittent aeration, the ammonia nitrogen content was decreased as the order: K5T15 (V1200) > K15T5 (V400) > K20T0 (V300) > K10T10 (V600) throughout the composting as the results of NH_3_ emission (*p* < 0.05).

Ammonia nitrogen is converted to nitrate nitrogen under the action of nitrifying bacteria, and nitrate nitrogen can be reduced to N_2_, NO, N_2_O, etc. under the action of denitrifying microorganisms (Shafiee-Jood and Cai, 2016). The nitrate nitrogen content of all groups gradually increased in composting (Figure 2D). Considering that the activity of nitrifying bacteria is easily affected by temperature (Xiong et al., 2023), nitrification is inhibited in the mesophilic and thermophilic stages, resulting in the mainly production of nitrate nitrogen in the later stage of composting. The nitrate nitrogen content of V150 with continuous aeration was higher than the other treatments on days 2 ~ 7 of composting and reached a maximum value of 5.33 g/kg by the end of composting. The nitrate nitrogen content of the treatments with aeration rate greater than 900 L/h was lower than that of V150 and V300 throughout the composting process, suggesting that the smaller aeration rate was favorable to the formation of nitrate nitrogen (*p* < 0.01). As for intermittent aeration, nitrate nitrogen content of K15T5 (V400), K10T10 (V600), and K5T15 (V1200) was higher than that of the continuous aeration K20T0 (V300) in composting (*p* > 0.05), indicating that under the premise of the same aeration volume in 20 min, the intermittent aeration favored the formation of nitrate nitrogen especially in the thermophilic stage.

The amide nitrogen content of all treatments showed a trend of increasing and then decreasing (Figure 2E). Similar to the variation pattern of ammonia nitrogen, amide nitrogen content of treatments with continuous aeration rates less than 600 L/h was higher than that with aeration rates greater than 900 L/h. The amide nitrogen of V150, V300, V600, V900, V1200, and V1500 peaked at 3.03, 3.48, 2.75, 1.41, 1.49, and 1.48 g/kg, respectively (*p* < 0.05). Composting on day 2–7 at the thermophilic stage had more amide nitrogen production. As for intermittent aeration, K5T15 (V1200) had the best effect to favor the formation of ammonia nitrogen, nitrate nitrogen, and amide nitrogen (*p* > 0.05).

The organic nitrogen content of each treatment showed a trend of decreasing and then increasing (Figure 2F). At the beginning of composting, the organic nitrogen decreased more in treatments with continuous aeration rates less than 600 L/h. V150, V300, and V600 reached the lowest values on day 4–6 with 0.98–1.20%. At the end of composting, the organic nitrogen content was about 1.80% for the three treatments with aeration rates less than 600 L/h, which was beneficial to the degradation of organic nitrogen (Zhang et al., 2016) (*p* < 0.05). As for intermittent aeration, the organic nitrogen content was about 1.90% at the end of composting. K5T15 (V1200) favored the degradation of organic nitrogen in the thermophilic stage and the degradation capacity of organic nitrogen in each treatment was decreased in the order: K5T15 (V1200) > K15T5 (V400) > K20T0 (V300) > K10T10 (V600) (*p* > 0.05).

The above results indicated that intermittent aeration mode by K5T15 (V1200) and V300 of continuous aeration helped to the conservation of nitrogen fractions and accelerate the composting process. However, considering the concentration effect of composting and the accumulated NH_3_ emission, it was most advantageous to use V150 of continuous aeration to reduce NH_3_ emission and ensure the effective composting process.

### 3.3 Dynamics of bacterial community

**Figure 3:**
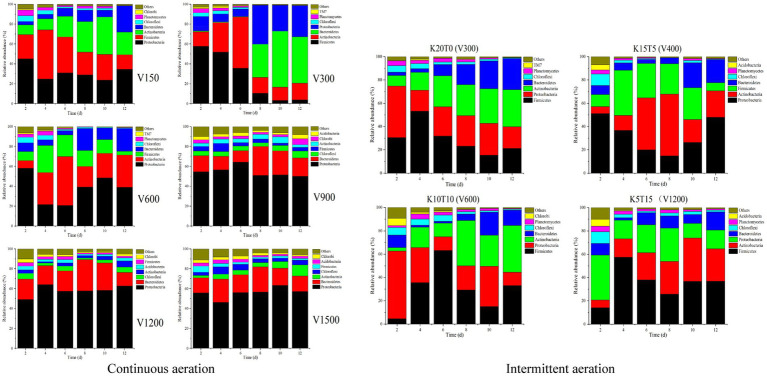
Relative abundance of bacterial phyla at different aeration rates of continuous (left) and intermittent (right) aeration mode during composting.

The variations in bacterial community composition at phylum level for different treatments during composting were shown in Figure 3. In groups with the aeration rate was less than 600 L/h of continuous aeration and all the groups with intermittent aeration, the dominant phyla were *Firmicutes*, *Actinobacteria*, and *Proteobacteria*, and the bacterial community composition changed significantly with the composting process. The relative abundance of *Firmicutes* were 9.00–57.62%, which was dominant in the mesophilic and thermophilic stages, due to its rapid growth under nutrient-rich conditions and high heat tolerance (Liu et al., 2024). The relative abundance of *Actinobacteria* and *Proteobacteria* were 7.87–51.39% and 7.44–58.01%, respectively, in the mesophilic and thermophilic stages. *Actinobacteria* and *Proteobacteria* are reported to be responsible for degrading cellulose, lignin, and proteins (Wei et al., 2019; Liu et al., 2021). When the aeration rate was greater than 900 L/h with continuous aeration, *Proteobacteria* and *Bacteroidetes* were the dominant phyla, and the bacterial community composition did not change significantly with the progress of composting. The members of *Bacteroidetes* are also involved in the degradation of lignocellulose and protein (Wei et al., 2019; Liu et al., 2021). The relative abundance of *Proteobacteria* were 46.36–64.19% during the composting. Before day 6, the relative abundance of *Proteobacteria* and *Firmicutes* was accounted for over 70% in total in V150 and V300 of continuous aeration and K20T0 (V300) with intermittent aeration. As the composting progressed, the relative abundance of *Actinobacteria* and *Bacteroidetes* gradually increased to nearly 50–60% by the end of composting in V150 and V300 with continuous aeration and K20T0 (V300) with intermittent aeration.

**Figure 4:**
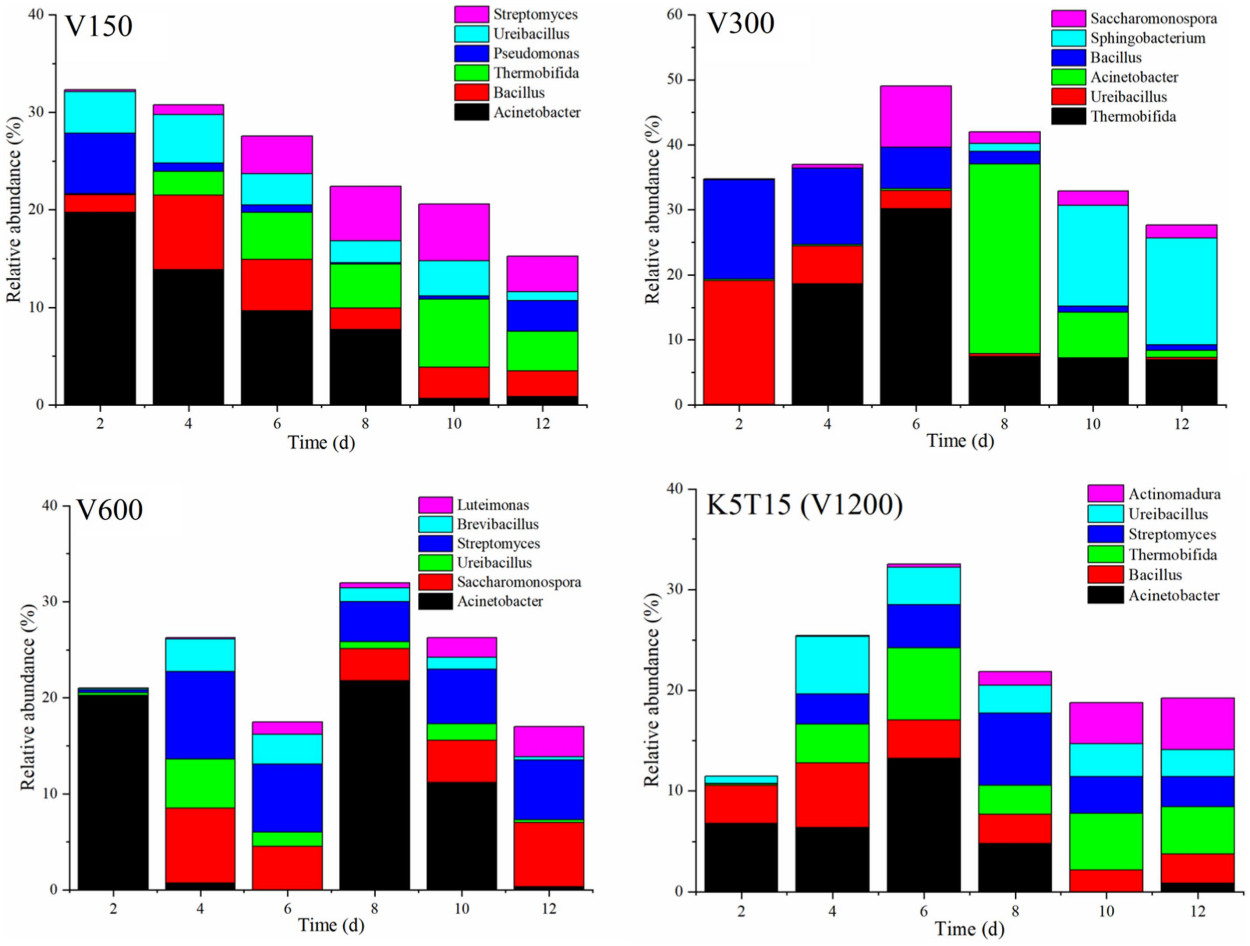
Relative abundance of bacterial genus in microbiota at different aeration modes and rates during composting. Continuous aeration: V150, V300, and V600. Intermittent aeration: K5T15 (V1200).

Considering that the better temperature rise, nitrogen fractions conversion and NH_3_ emission reduction, as well as longer duration of thermophilic stage in V150, V300, and V600 of continuous aeration and K5T15 (V1200) of intermittent aeration, we further analyzed the variations in main bacterial genera in these groups (Figure 4; Table 2). The dominant genera of V150 were *Acinetobacter*, *Bacillus*, *Thermobifida*, *Pseudomonas*, *Ureibacillus*, *Streptomyces*, etc. with *Acinetobacter* having the highest relative abundance (19.72%) and gradually decreasing in composting. The relative abundance of *Bacillus* (7.68%) and *Ureibacillus* (4.98%) was higher in the early stage of composting, and the relative abundance of *Thermobifida* (6.95%) and *Streptomyces* (5.80%) was higher in the late stage of composting. The dominant genera of V300 were *Thermobifida*, *Ureibacillus*, *Acinetobacter*, *Bacillus*, *Sphingobacterium*, and *Saccharomonospora*, etc. and the relative abundance of *Thermobifida*, *Acinetobacter*, and *Saccharomonospora* was 30.16, 29.18, and 9.40% in the thermophilic stage of composting due to their high temperature tolerance. The dominant genera of V600 were *Acinetobacter*, *Streptomyces*, and *Saccharomonospora*. *Saccharomonospora* at the end composting had higher relative abundance (6.69%) than other genera. The dominant genera in K5T15 (V1200) were *Acinetobacter*, *Bacillus*, *Thermobifida*, *Streptomyces*, *Ureibacillus*, and *Actinomadura*. These results showed that there was an obvious increase of relative abundance of *Thermobifida* in V300 and K5T15 (V1200), suggesting that *Thermobifida* as core bacteria had significant positive effect on composting process (Zhan et al., 2021). *Pseudomonas* had an obvious advantage in V150 compared to other groups, which was reported to be nitrifiers with *amoA* gene and denitrifiers with nitrite reductase genes and nitrous oxide reductase (Hoang et al., 2022).

**Table 2:** The relative abundances of major genera during the composting.

Treatment	Day	Acinetobacter (%)	Bacillus (%)	Thermobifida (%)	Pseudomonas (%)	Ureibacillus (%)	Streptomyces (%)
V150	2	19.72	1.80	0.13	6.21	4.27	0.18
	4	13.83	7.68	2.41	0.84	4.98	1.02
	6	9.62	5.28	4.83	0.76	3.21	3.87
	8	7.74	2.19	4.48	0.15	2.24	5.59
	10	0.66	3.21	6.95	0.35	3.60	5.80
	12	0.86	2.60	4.09	3.15	0.90	3.63
V300		**Acinetobacter (%)**	**Bacillus (%)**	**Thermobifida (%)**	**Ureibacillus (%)**	**Sphingobacterium (%)**	**Saccharomonospora (%)**
	2	0.18	15.33	0.04	19.09	0	0.12
	4	0.22	11.74	18.62	5.80	0	0.60
	6	0.32	6.39	30.16	2.78	0	9.40
	8	29.18	1.96	7.43	0.43	1.18	1.78
	10	7.03	0.95	7.08	0.11	15.48	2.25
	12	1.08	0.87	6.91	0.40	16.35	2.03
V600		**Acinetobacter (%)**	**Ureibacillus (%)**	**Luteimonas (%)**	**Streptomyces (%)**	**Brevibacillus (%)**	**Saccharomonospora (%)**
	2	20.26	0.28	0.05	0.30	0.15	0
	4	0.71	5.14	0.12	9.12	3.40	7.78
	6	0	1.51	1.32	7.07	3.07	4.51
	8	21.76	0.71	0.53	4.14	1.47	3.36
	10	11.17	1.70	2.05	5.71	1.23	4.40
	12	0.31	0.30	3.19	6.21	0.32	6.69
K5T15 (V1200)		**Acinetobacter (%)**	**Bacillus (%)**	**Thermobifida (%)**	**Streptomyces (%)**	**Ureibacillus (%)**	**Actinomadura (%)**
	2	6.80	3.78	0.11	0.08	0.69	0
	4	6.40	6.40	3.86	2.99	5.69	0.09
	6	13.25	3.79	7.16	4.30	3.70	0.30
	8	4.80	2.89	2.86	7.159	2.77	1.39
	10	0	2.19	5.60	3.65	3.25	4.08
	12	0.86	2.90	4.66	2.99	2.68	5.13

## 4 Conclusion

The continuous aeration mode V300 and intermittent aeration mode K5T15 (V1200) had a better effect in terms of temperature rise, GI value, and nitrogen fractions conversion compared to other aeration rates in composting of dehydrated sludge and corn straw. In a comprehensive comparison, the intermittent aeration mode was superior to the continuous aeration mode at same aeration volume, especially in terms of NH_3_ emission reduction and GI with the highest value (94.65%) in K5T15 (V1200). The relative abundance of *Firmicutes* was greater in the intermittent aeration mode than that in the continuous aeration mode. *Thermobifida* was the core bacteria for significantly accelerating composting process and *Pseudomonas* was dominant in V150 with the lowest NH_3_ emission.

## Data availability statement

The raw data supporting the conclusions of this article will be made available by the authors, without undue reservation.

## Author contributions

YuyW: Conceptualization, Data curation, Writing – original draft. PX: Conceptualization, Data curation, Investigation, Methodology, Resources, Writing – original draft. YueW: Conceptualization, Writing – original draft. JS: Data curation, Writing – original draft. ZX: Resources, Writing – review & editing. ZJ: Data curation, Writing – original draft. YuqW: Funding acquisition, Project administration, Writing – review & editing. SH: Conceptualization, Formal analysis, Writing – original draft. XD: Formal analysis, Methodology, Writing – original draft. HZ: Resources, Visualization, Writing – original draft. LZ: Resources, Validation, Writing – original draft. YL: Methodology, Software, Visualization, Writing – original draft, Writing – review & editing. JL: Resources, Writing – review & editing.

## Footnotes

^1^http://www.freebioinfo.org

## References

[ref1] Caceres R. Magri A. Marfa O. (2015). Nitrification of leachates from manure composting under field conditions and their use in horticulture. Waste Manag. 44, 72–81. doi: 10.1016/j.wasman.2015.07.039, PMID: 26239938

[ref2] Chowdhury M. A. de Neergaard A. Jensen L. S. (2014). Potential of aeration flow rate and bio-char addition to reduce greenhouse gas and ammonia emissions during manure composting. Chemosphere 97, 16–25. doi: 10.1016/j.chemosphere.2013.10.030, PMID: 24210550

[ref3] Gao M. C. Li B. Yu A. Liang F. Y. Yang L. J. Sun Y. X. (2010). The effect of aeration rate on forced-aeration composting of chicken manure and sawdust. Bioresour. Technol. 101, 1899–1903. doi: 10.1016/j.biortech.2009.10.027, PMID: 19897360

[ref4] Gao X. Tan W. Zhao Y. Wu J. Sun Q. Qi H.. (2019). Diversity in the mechanisms of humin formation during composting with different materials. Environ. Sci. Technol. 53, 3653–3662. doi: 10.1021/acs.est.8b06401, PMID: 30821974

[ref5] Han Z. L. Sun D. Z. Wang H. Li R. Y. Bao Z. Y. Qi F. (2018). Effects of ambient temperature and aeration frequency on emissions of ammonia and greenhouse gases from a sewage sludge aerobic composting plant. Bioresour. Technol. 270, 457–466. doi: 10.1016/j.biortech.2018.09.048, PMID: 30245315

[ref6] Hoang H. G. Thuy B. T. P. Lin C. Vo D. V. N. Tran H. T. Bahari M. B.. (2022). The nitrogen cycle and mitigation strategies for nitrogen loss during organic waste composting: a review. Chemosphere 300:134514. doi: 10.1016/j.chemosphere.2022.134514, PMID: 35398076

[ref7] Jiang T. Li G. Tang Q. Ma X. Wang G. Schuchardt F. (2015). Effects of aeration method and aeration rate on greenhouse gas emissions during composting of pig feces in pilot scale. J. Environ. Sci. 31, 124–132. doi: 10.1016/j.jes.2014.12.005, PMID: 25968266

[ref8] Keener H. M. Elwell D. L. Ekinci K. Hoitink H. A. J. (2001). Composting and value-added utilization of manure from a swine finishing facility. Compost Sci. Util. 9, 312–321. doi: 10.1080/1065657X.2001.10702050

[ref9] Lai J. C. Then Y. L. San Hwang S. Lee C. S. (2024). Optimal aeration management strategy for a small-scale food waste composting. Carbon Resour. Conv. 7:100190. doi: 10.1016/j.crcon.2023.06.002

[ref10] Lehtovirta-Morley L. E. Verhamme D. T. Nicol G. W. Prosser J. I. (2013). Effect of nitrification inhibitors on the growth and activity of Nitrosotalea devanaterra in culture and soil. Soil Biol. Biochem. 62, 129–133. doi: 10.1016/j.soilbio.2013.01.020

[ref11] Liu Y. D. Qian Y. L. Yong X. Y. Jia H. H. Wei P. Zhou J. (2021). Effects of granular activated carbon and temperature on the viscosity and methane yield of anaerobically digested of corn straw with different dry matter concentrations. Bioresour. Technol. 332:125109. doi: 10.1016/j.biortech.2021.125109, PMID: 33839508

[ref12] Liu Y. Wang H. Zhang H. Tao Y. Chen R. Hang S.. (2024). Synergistic effects of chemical additives and mature compost on reducing H_2_S emission during kitchen waste composting. J. Environ. Sci. 139, 84–92. doi: 10.1016/j.jes.2023.05.030, PMID: 38105080

[ref13] Manu M. K. Li D. Liwen L. Jun Z. Varjani S. Wong J. W. (2021). A review on nitrogen dynamics and mitigation strategies of food waste digestate composting. Bioresour. Technol. 334:125032. doi: 10.1016/j.biortech.2021.125032, PMID: 33964812

[ref14] Meng L. Q. Li W. G. Zhang S. M. Wu C. D. Wang K. (2016). Effects of sucrose amendment on ammonia assimilation during sewage sludge composting. Bioresour. Technol. 210, 160–166. doi: 10.1016/j.biortech.2016.01.094, PMID: 26852272

[ref15] Mu D. Mu L. Geng X. Mohamed T. A. Wei Z. (2024). Evolution from basic to advanced structure of fulvic acid and humic acid prepared by food waste. Int. J. Biol. Macromol. 256:128413. doi: 10.1016/j.ijbiomac.2023.128413, PMID: 38029895

[ref16] Onwosi C. O. Igbokwe V. C. Odimba J. N. Eke I. E. Nwankwoala M. O. Iroh I. N.. (2017). Composting technology in waste stabilization: on the methods, challenges and future prospects. J. Environ. Manag. 190, 140–157. doi: 10.1016/j.jenvman.2016.12.051, PMID: 28040590

[ref17] Peng L. Tang R. Wang G. Ma R. Li Y. Li G.. (2023). Effect of aeration rate, aeration pattern, and turning frequency on maturity and gaseous emissions during kitchen waste composting. Environ. Technol. Innov. 29:102997. doi: 10.1016/j.eti.2022.102997

[ref18] Rasapoor M. Nasrabadi T. Kamali M. Hoveidi H. (2009). The effects of aeration rates on generated compost quality, using aerated static pile method. Waste Manag. 29, 570–573. doi: 10.1016/j.wasman.2008.04.012, PMID: 18619830

[ref19] Shafiee-Jood M. Cai X. M. (2016). Reducing food loss and waste to enhance food security and environmental sustainability. Environ. Sci. Technol. 50, 8432–8443. doi: 10.1021/acs.est.6b01993, PMID: 27428555

[ref20] Shan G. Li W. Gao Y. Tan W. Xi B. (2021). Additives for reducing nitrogen loss during composting: a review. J. Clean. Prod. 307:127308. doi: 10.1016/j.jclepro.2021.127308

[ref21] Sheng Y. Baars O. Guo D. Whitham J. Srivastava S. Dong H. (2023). Mineral-bound trace metals as cofactors for anaerobic biological nitrogen fixation. Environ. Sci. Technol. 57, 7206–7216. doi: 10.1021/acs.est.3c01371, PMID: 37116091

[ref22] Talib A. T. Mokhtar M. N. Baharuddin A. S. Sulaiman A. (2014). Effects of aeration rate on degradation process of oil palm empty fruit bunch with kinetic-dynamic modeling. Bioresour. Technol. 169, 428–438. doi: 10.1016/j.biortech.2014.07.033, PMID: 25079208

[ref01] Wang Y. Tang Y. Li M. Yuan Z. (2021). Aeration rate improves the compost quality of food waste and promotes the decomposition of toxic materials in leachate by changing the bacterial community. Bioresource Tec. 340:125716. 10.1016/j.biortech.2021.125716 34385125

[ref23] Wei Y. Wu D. Wei D. Zhao Y. Wu J. Xie X.. (2019). Improved lignocellulose-degrading performance during straw composting from diverse sources with actinomycetes inoculation by regulating the key enzyme activities. Bioresour. Technol. 271, 66–74. doi: 10.1016/j.biortech.2018.09.081, PMID: 30265954

[ref24] Wei Y. Zhao Y. Shi M. Cao Z. Lu Q. Yang T.. (2018). Effect of organic acids production and bacterial community on the possible mechanism of phosphorus solubilization during composting with enriched phosphate-solubilizing bacteria inoculation. Bioresour. Technol. 247, 190–199. doi: 10.1016/j.biortech.2017.09.092, PMID: 28950126

[ref25] Wei Y. Zhao Y. Zhao X. Gao X. Zheng Y. Zuo H.. (2020). Roles of different humin and heavy-metal resistant bacteria from composting on heavy metal removal. Bioresour. Technol. 296:122375. doi: 10.1016/j.biortech.2019.122375, PMID: 31734063

[ref26] Wu D. Gao W. Zhao Y. Wei Z. Song C. Qu F.. (2024). Elaborating the microbial mechanism of humic substance formation during lignocellulosic biomass composting by inoculation with different functional microbes. Ind. Crop. Prod. 208:117838. doi: 10.1016/j.indcrop.2023.117838

[ref27] Wu J. Zhao Y. Yu H. Wei D. Yang T. Wei Z.. (2019). Effects of aeration rates on the structural changes in humic substance during co-composting of digestates and chicken manure. Sci. Total Environ. 658, 510–520. doi: 10.1016/j.scitotenv.2018.12.198, PMID: 30579208

[ref28] Xin L. Qin Y. Lou T. Xu X. Wang H. Mei Q.. (2023). Rapid start-up and humification of kitchen waste composting by an innovative biodrying-enhanced process. Chem. Eng. J. 452:139459. doi: 10.1016/j.cej.2022.139459

[ref29] Xiong S. Liu Y. Zhang H. Xu S. Li S. Fan X.. (2023). Effects of chemical additives and mature compost on reducing nitrogen loss during food waste composting. Environ. Sci. Pollut. Res. 30, 39000–39011. doi: 10.1007/s11356-022-24752-5, PMID: 36593319

[ref30] Xiong Z. Q. Wang G. X. Huo Z. C. Yan L. Gao Y. M. Wang Y. J.. (2017). Effect of aeration rates on the composting processes and nitrogen loss during composting. Appl. Environ. Biotechnol. 2, 20–27. doi: 10.26789/AEB.2017.01.003

[ref31] Zhan Y. Zhang Z. Ma T. Zhang X. Wang R. Liu Y.. (2021). Phosphorus excess changes rock phosphate solubilization level and bacterial community mediating phosphorus fractions mobilization during composting. Bioresour. Technol. 337:125433. doi: 10.1016/j.biortech.2021.125433, PMID: 34171708

[ref32] Zhang H. Y. Li G. X. Gu J. Wang G. Q. Li Y. Y. Zhang D. F. (2016). Influence of aeration on volatile sulfur compounds (VSCs) and NH3 emissions during aerobic composting of kitchen waste. Waste Manag. 58, 369–375. doi: 10.1016/j.wasman.2016.08.022, PMID: 27595496

[ref33] Zhao L. Zhao M. Gao W. Xie L. Zhang G. Li J.. (2024). Different Bacillus sp. play different roles on humic acid during lignocellulosic biomass composting. J. Clean. Prod. 434:139901. doi: 10.1016/j.jclepro.2023.139901

[ref34] Zhou A. Wang X. B. Yu S. L. Deng S. H. Tan H. Z. Mikulcic H. (2023). Process design and optimization on self-sustaining pyrolysis and carbonization of municipal sewage sludge. Waste Manag. 159, 125–133. doi: 10.1016/j.wasman.2023.01.035, PMID: 36753855

